# Risk-Based Admission Screening for Candida auris and Carbapenemase-Producing Organisms within an Acute Care Health System

**DOI:** 10.1017/ash.2025.424

**Published:** 2025-09-24

**Authors:** Joan Ivaska, Aarikha D’Souza, Morgan Baker, Steven Watson, Alice Hughes, William Lainhart

**Affiliations:** 1 Banner Health; 2 Banner Health; 3 Banner Health; 4 Banner Health; 5 Banner Health; 6 Banner Health/University of Arizona

## Abstract

**Background:** Patients infected or colonized with Candida auris or Carbapenemase-Producing Organisms (CPO) can serve as a transmission source for other patients. Screening select patients for these organisms allows facilities to implement infection prevention measures to minimize the risk of transmission.

Following a two-year period of ongoing transmission of Candida auris occurring in a community served by a large multi-state healthcare system, and the passing of a board of health rule requiring admission screening of select patients in another community served by the health system, an admission screening program was implemented across all acute care facilities in the system in 2024. **Methods:** Beginning in March of 2024, all patient admissions were screened for history of an overnight stay in either a long-term acute care (LTAC), skilled nursing (SNF), or non-US healthcare facility, or having had an invasive medial or surgical procedure outside of the US in the past 12 months. Patients who screened positive were placed in transmission-based precautions and consented for screening for Candida auris and CPO. Screening tests were performed utilizing a two-step method of HardyCHROM agar followed by UV fluorescence, MALDI-TOF MS or CARBA5. Potentially exposed patients who had been discharged were not screened. **Results:** 4249 patients in the acute care facilities were identified for admission screening, with 2553 consented and screened for *Candida auris* and 3346 for CPO respectively. Admission screening positivity rates are in table 1. No Risk Factors With Risk Factors Overall Candida auris 2.3% 2.3% 2.3% CPO 6.5% 7.25% 7.11%. Table 1: Admission screening positivity rates by pathogen and risk factors **Conclusion:** Candida auris colonization rates were the same in patients with and without risk factors. CPO colonization rates were 11% higher in patients with a risk factor present. 96.4% of the CPO positive screens had a LTAC/SNF stay as their risk factor. More robust surveillance and prevention strategies are needed in these care settings to prevent CPOs endemicity. Limitations of this study include the lack of screening completion in discharged patients and those patients who did not consent.

